# Interleukin-7 enhances *in vitro* development and blastocyst quality in porcine parthenogenetic embryos

**DOI:** 10.3389/fvets.2022.1052856

**Published:** 2022-12-08

**Authors:** Dongjin Oh, Hyerin Choi, Mirae Kim, Lian Cai, Joohyeong Lee, Ali Jawad, Sohee Kim, Haomiao Zheng, Gabsang Lee, Yubyeol Jeon, Sang-Hwan Hyun

**Affiliations:** ^1^Laboratory of Veterinary Embryology and Biotechnology (VETEMBIO), Veterinary Medical Center and College of Veterinary Medicine, Chungbuk National University, Cheongju, South Korea; ^2^Institute of Stem Cell and Regenerative Medicine (ISCRM), Chungbuk National University, Cheongju, South Korea; ^3^Graduate School of Veterinary Biosecurity and Protection, Chungbuk National University, Cheongju, South Korea; ^4^Department of Neurology, Institute for Cell Engineering, School of Medicine, Johns Hopkins Medicine, Baltimore, ML, United States; ^5^Laboratory of Theriogenology and Reproductive Biotechnology, College of Veterinary Medicine, Jeonbuk National University, Iksan, South Korea

**Keywords:** interleukin-7, pig, embryonic development, *in vitro* culture, parthenogenetic activation

## Abstract

Interleukin-7 (IL-7), a vital factor that affects cell development, proliferation, and survival, plays an important role in oocyte maturation. However, its role in embryonic development remains unknown. Therefore, in this study, we aimed to investigate the effects of IL-7 supplementation on *in vitro* culture (IVC) of porcine embryos after parthenogenetic activation (PA) based on characteristics such as cleavage, blastocyst formation rate, intracellular glutathione (GSH) and reactive oxygen species (ROS) levels in cleaved embryos, total cell number, apoptosis rate, and cell lineage specification in blastocysts. Immunofluorescence revealed that IL-7 and its receptor, IL-7Rα (IL-7R) localized in the cytoplasm of porcine parthenote embryos. By supplementing the IVC medium (PZM5) with various concentrations of IL-7, an optimal concentration that enhanced embryonic development, promoted intracellular GSH, and decreased ROS levels in the cleavage stage during porcine embryo IVC was determined. Investigation of mRNA expression patterns *via* qRT-PCR suggested that IL-7 possibly regulated maternal mRNA clearance and zygotic genome activation. Furthermore, IL-7 supplementation reduced blastocyst apoptosis, enhanced the expression of the inner cell mass marker SOX2, and phosphorylated STAT5 levels in the blastocysts. Moreover, it altered the transcription patterns of genes that regulate apoptosis, IL-7 signaling, and development. Thus, we demonstrated the localization of IL-7 and IL-7R in porcine preimplantation embryos *in vitro* for the first time. Furthermore, we suggest that IL-7 supplementation can be employed to enhance embryonic development and blastocyst quality based on the activation of the transcripts of genes that are involved in developmental competence and IL-7 signaling during *in vitro* porcine embryo development following PA.

## Introduction

Assisted reproductive technologies (ARTs), such as *in vitro* fertilization (IVF) and embryo cryopreservation facilitate conception in domestic animals with fertility issues. Embryo transfer is the most critical step in IVF, and its success rate depends on several factors, including embryo quality and the technique used (1). In livestock and veterinary medicine, the pig industry is considerably important, with pig productivity management being a key aspect in the latter category. Given that the preservation of genetic resources in breeding pigs and the international trade of fertilized embryos have become active, the use of ARTs such as *in vitro* culture (IVC), and cryopreservation of fertilized embryos derived from breeding pigs has increased rapidly (2, 3).

Pigs serve as useful models in several research areas, such as reproductive biotechnology, transgenesis, and biomedicine. They can also serve as suitable organ donors in regenerative medicine and human disease models owing to the genetic, anatomical, and physiological similarities between them and humans (4, 5). For example, embryonic stem cells (ESCs) derived from pig blastocysts are powerful tools for preliminary studies on human diseases (6), and to obtain ESCs from blastocysts cultured *in vitro*, blastocyst quality is an important factor to take into consideration.

In several studies, various components, such as antioxidants, growth factors, and hormones, have been added to the embryo culture medium to increase the likelihood of obtaining high-quality embryos *in vitro* (7–9). Specifically, cytokines act as regulators of ovarian physiology, creating an immune-permissive embryonic environment that encourages gametogenesis, fertilization, early embryonic development, blastocyst hatching and implantation, and fetal growth (10–14). The reproductive role of interleukins (ILs) as cytokines has been demonstrated in several studies. For example, ILs such as IL-1β, IL-6, and IL-8 interact with oocytes and their surrounding somatic cells during folliculogenesis (15). Although several IL families have been identified, their functional roles in embryonic development have not yet been sufficiently clarified.

IL-7, which is a member of the IL-2 superfamily, plays an essential role in the survival and development of *T* and *B* cells of the immune system (16). Further, it signals by binding to the IL-7 receptor complex, which comprises IL-7Rα (IL-7R) and the common cytokine receptor γ-chain (IL-2RG) (17). It also regulates anti-apoptotic and proliferative signals *via* the Janus kinase–signal transducer and activator of transcription (JAK-STAT) and phosphoinositide 3-kinase (PI3K) pathways. Moreover, it modulates B-cell lymphoma 2 (BCL2) family members (18). In the reproductive system, IL-7 has been detected in both human (19, 20) and porcine follicular fluid (FF) (21), and its functional roles in oocytes and surrounding somatic cells in various species have been reported (22). Additionally, it has been observed that IL-7 treatment modulates the phosphorylation of AKT and STAT5 proteins and inhibits apoptosis in rat granulosa cells (23). However, the functional role of IL-7 in the embryonic development of domestic animals remains unknown and requires further investigation.

In the present study, to clarify the functional role of IL-7 during embryonic IVC, we first investigated the presence of IL-7 and IL-7R proteins in preimplantation porcine embryos *via* immunostaining. Thereafter, we explored the effects of IL-7 supplementation during IVC on embryonic development, intracellular glutathione (GSH) levels, reactive oxygen species (ROS) levels, and apoptosis in porcine parthenote embryos cultured *in vitro*. The mRNA expression levels of specific genes in parthenogenetic activation (PA)-derived cleavage and blastocysts treated with IL-7 were examined by quantitative reverse transcription-polymerase chain reaction (qRT-PCR). Finally, the expression of the inner cell mass (ICM) marker (SOX2) and phosphorylated STAT5 (pSTAT5), which are related to IL-7 signaling, were confirmed *via* immunostaining. We believe that our study contributes important information that can be used to improve porcine blastocyst quality during IVC.

## Materials and methods

### Chemicals and reagents

Recombinant human IL-7 was purchased from PeproTech (London, UK). Unless otherwise indicated, all the chemicals and reagents used in this study were obtained from Sigma-Aldrich (St. Louis, MO, USA).

### Oocyte collection and *in vitro* maturation

Porcine ovaries were collected from a local abattoir and transported to the laboratory within 1 h in 0.9% (v/v) NaCl solution at 37°C. At the laboratory, the ovaries were washed twice with 0.9% (v/v) NaCl solution, and thereafter, cumulus-oocyte complexes (COCs) were retrieved from medium sized (3–7 mm in diameter) follicles (24) using an 18-gauge needle and a 10 mL disposable syringe and collected into 15 mL conical tubes. The COCs were washed with HEPES-buffered Tyrode's medium containing 0.05% (w/v) polyvinyl alcohol (TLH-PVA). Then, 50–60 COCs with compact cumulus cell layers and evenly granulated cytoplasm were cultured in a four-well dish (Nunc, Roskilde, and Denmark) containing 500 μL IVM medium, consisting of TCM-199 (Invitrogen Corporation, Carlsbad, CA, USA) supplemented with 0.6 mM cysteine, 0.91 mM sodium pyruvate, 10 ng/mL epidermal growth factor, 75 μg/mL kanamycin, 1 μg/mL insulin, and 10% (v/v) porcine FF. The IVM process took place over a period of 42 h. This was followed by the incubation of the COCs with 10 IU/mL equine chorionic gonadotropin and 10 IU/mL human chorionic gonadotropin at 39°C in a humidified 5% CO_2_ atmosphere first for 22 h, after which the COCs were then transferred to a hormone-free IVM medium and further incubated for the remaining 20 h.

### Parthenogenetic activation and *in vitro* culture of porcine embryos

PA was performed according to a previously reported protocol (25). Briefly, after IVM, metaphase II—stage (MII) oocytes were obtained by mechanically pipetting enclosing cumulus cells in the presence of 0.1% hyaluronidase for 1 min. After washing twice with the activation solution (280 mM mannitol solution containing 0.01 mM CaCl_2_ and 0.05 mM MgCl_2_), the MII oocytes were activated in 2 mL of the same solution with two direct electrical pulses of 120 V/mm for 60 μs, using a cell fusion generator (LF101; NepaGene, Chiba, Japan). Thereafter, the oocytes were cultured in the IVC medium (porcine zygote medium 5; PZM5) (26) containing 5 μg/mL of cytochalasin B for 4 h in a humidified atmosphere of 5% CO_2_, 5% O_2_, and 95% N_2_. The electrically activated embryos then were washed three times in droplets of IVC medium (30 μL) and incubated in the same droplets (10 embryos per droplet) covered with mineral oil. PA was carried out on day 0. The embryos were moved to fresh droplets 48 (day 2) and 96 h (day 4) after PA. On day 2, the embryo cleavage rates were analyzed in five categories (1-, 2–3-, 4–5-, 6–8-cell, and fragmented embryos). On day 7, embryonic development was quantitatively evaluated by determining the cleavage and blastocyst formation rates in three groups, according to blastocyst morphology (early, expanded, and hatched). During the entire IVC period, IL-7 was added to the IVC media at concentrations of 0 (control), 0.1, 1, 10, and 100 ng/mL.

### Measurement of intracellular GSH and ROS levels

Intracellular GSH and ROS levels were measured using a previously reported method (27). The 4 to 5-cell stage embryos in each group were sampled on day 2 to make these measurements. CellTracker Blue 4-chloromethyl-6,8-difluoro-7-hydroxycoumarin (CMF_2_HC; Invitrogen) and 2'7'-dichlorodihydrofluorescein diacetate (H_2_DCFDA; Invitrogen) were used to measure intracellular GSH (blue fluorescence) and ROS (green fluorescence) levels in the embryo cytoplasm. In brief, the embryos were transferred to TLH-PVA medium containing 10 μM CMF_2_HC or H_2_DCFDA and stained in the dark for 30 and 10 min, respectively. Thereafter, the embryos were washed three times with TLH-PVA and transferred to 8 μL droplets of TLH-PVA. GSH and ROS levels were then imaged using a fluorescence microscope (TE300; Nikon, Tokyo, Japan) with UV filters (370 nm for GSH and 460 nm for ROS). Finally, Adobe Photoshop 2021 was used to analyze the fluorescence intensity of each embryo, which was normalized to that of the control group.

### Terminal deoxynucleotidyl transferase-mediated dUTP nick end labeling assay

Blastocysts were stained with TUNEL to determine the number of apoptotic cells using an *in situ* cell death detection kit (Roche, Mannheim, Germany), according to a previously reported method (28). On day 7, the blastocysts derived from the IVC medium were treated with 0, 0.1, 1, 10, and 100 ng/mL of IL-7, washed three times in 0.1% PBS-PVA (PVS), and fixed in 4% paraformaldehyde in PBS for 30 min at 25°C (room temperature; RT). In the next step, the blastocysts were washed twice in 0.1% PVS with 0.1% Tween 20 and 0.01% Triton X-100 (v/v) and permeabilized with 0.3% TritonX-100 in PBS for 1 h at 37°C. Finally, TUNEL assay was performed using fluorescein-conjugated deoxyuridine triphosphate (dUTP) and terminal deoxynucleotidyl transferase (Roche, Mannheim, Germany) for 1 h 30 min at 37°C. After washing twice in 0.1% PVS, the blastocysts were counterstained with 5 μg/mL Hoechst-33342 for 10 min at RT to visualize nuclei.

### Quantitative reverse transcription-polymerase chain reaction

The mRNA expression was analyzed using qRT-PCR for 19 specific genes associated with different functions, such as apoptosis-related genes: BCL2 associated X (*BAX*), BCL2 like 1 (*BCL2L1*), caspase-3 (*CASP3*), and myeloid cell leukemia-1 (*MCL1*); IL-7 signaling-related genes: phosphoinositide-3-kinase regulatory subunit 1 (*PIK3R1*), AKT serine/threonine kinase 1 (*AKT1*), and extracellular-regulated kinase 1/2 (*ERK1/2*; also known as *MAPK3/1*); embryonic development-related genes: proliferating cell nuclear antigen (*PCNA*), POU class 5 homeobox 1 (*OCT4*), *NANOG*, caudal type homeobox 2 (*CDX2*), and GATA binding protein 6 (*GATA6*); maternal effect genes (MEGs): KH domain containing 3 like (*KHDC3L*; *Filia*), nucleophosmin/nucleoplasmin 2 (*NPM2*), and zygote arrest 1 (*ZAR1*); zygotic genome activation (ZGA)–related genes: solute carrier family 34 member 2 (*SLC34A2*), developmental pluripotency associated 2 (*DPPA2*), and eukaryotic translation initiation factor 1A (*EIF1A*). All primer sequences are listed in Supplementary Table 1.

On day 2, embryos and blastocysts were washed thrice with PVS and sampled at −80°C before the experiment. RNA extraction was performed using TRIzol reagent (TaKaRa Bio, Inc., Otsu, Shiga, Japan), according to the manufacturer's protocol. Then, the extracted RNA (1 μg of total RNA) was converted to complementary DNA (cDNA) using SuperScript IV VILO Master Mix (Thermo Fisher Scientific, MA, USA). Finally, the synthesized cDNA, 2× SYBR Premix Ex Taq (TaKaRa Bio, Inc.), and 5 pmol of specific primers (Macrogen, Inc., Seoul, Republic of Korea) were used for qRT-PCR. The mRNA expression was analyzed using the CFX96 Touch Real-Time PCR Detection System (Bio-Rad, Hercules, CA, USA). The cycling parameters were as follows: 95°C for 5 min, followed by 40 cycles of 95°C for 15 s, 56°C for 15 s, and 72°C for 30 s. Relative quantification was performed by comparing the threshold cycle (Ct) at constant fluorescence intensity. Relative mRNA expression (R) was calculated using the equation *R* = 2^−[ΔCtsample−ΔCtcontrol]^ (29). The R values were normalized to those of *RN18S* in the blastocysts of each group.

### Immunofluorescence

Immunofluorescence was conducted according to the methods of Yoon et al. (8), with a few modifications. Briefly, the MII oocytes and embryos were fixed with 4% paraformaldehyde in PBS for 30 min at RT, permeabilized in 0.5% Triton X-100 for 1 h at RT and washed twice with 0.1% PVS. The MII oocytes or embryos were treated with Image-iT™ FX Signal Enhancer (Invitrogen, Carlsbad, CA, USA) for 30 min at RT and blocked in PBS with 3% BSA and 0.05% Tween 20, for 1 h 30 min at RT. The samples were then incubated overnight at 4°C with the following primary antibodies: rabbit anti-IL-7 (ab 175380, 1:50 dilution in blocking solution; Abcam, Cambridge, UK), rabbit anti-IL-7R (ab 180521, 1:100; Abcam), mouse anti-SOX2 (sc-365823, 1:100; Santa Cruz Biotechnology, Santa Cruz, CA, USA), and mouse anti-phospho-STAT5 (ab 106095, 1:25; Abcam). On the following day, the samples were washed three times for 5 min with 0.1% Tween 20 and 0.01% Triton X-100 in 0.1% PVS (TTVS) at RT and then incubated with the appropriate secondary antibodies: goat anti-mouse IgG (H + L) Alexa Fluor^TM^ 488 (A11029, 1:200; Invitrogen Corporation, Carlsbad, CA, USA), donkey anti-rabbit IgG (H + L) Alexa Fluor^TM^ 594 (A21207, 1:400; Invitrogen) for 2 h at RT. After washing three times in TTVS, the samples were counterstained with Hoechst-33342 for 10 min and mounted on glass slides in an anti-fade mounting medium (Molecular Probes, Inc., Eugene, OR, USA). The stained samples were analyzed using an epifluorescence microscope (TE300; Nikon) with UV filters. SOX2-positive cells were specifically examined in blastocysts with more than 30 cells on day 7.

### Statistical analysis

Statistical analysis was performed using SPSS 21.0 (SPSS Inc., Chicago, IL, USA). The experiments were performed at least in triplicates unless stated otherwise. Further, the results were presented as the mean ± SEM. Percentage data (cleavage and blastocyst formation rates) and average data (intracellular GSH and ROS levels in cleavage, TUNEL assay in blastocysts, relative gene expression levels, and quantitative analysis in immunofluorescence) were analyzed using one-way analysis of variance or Student's *t*-test. Statistical significance was set at *p* < 0.05.

## Results

### Localization of IL-7 and IL-7R proteins in porcine preimplantation embryos

**Figure 1:**
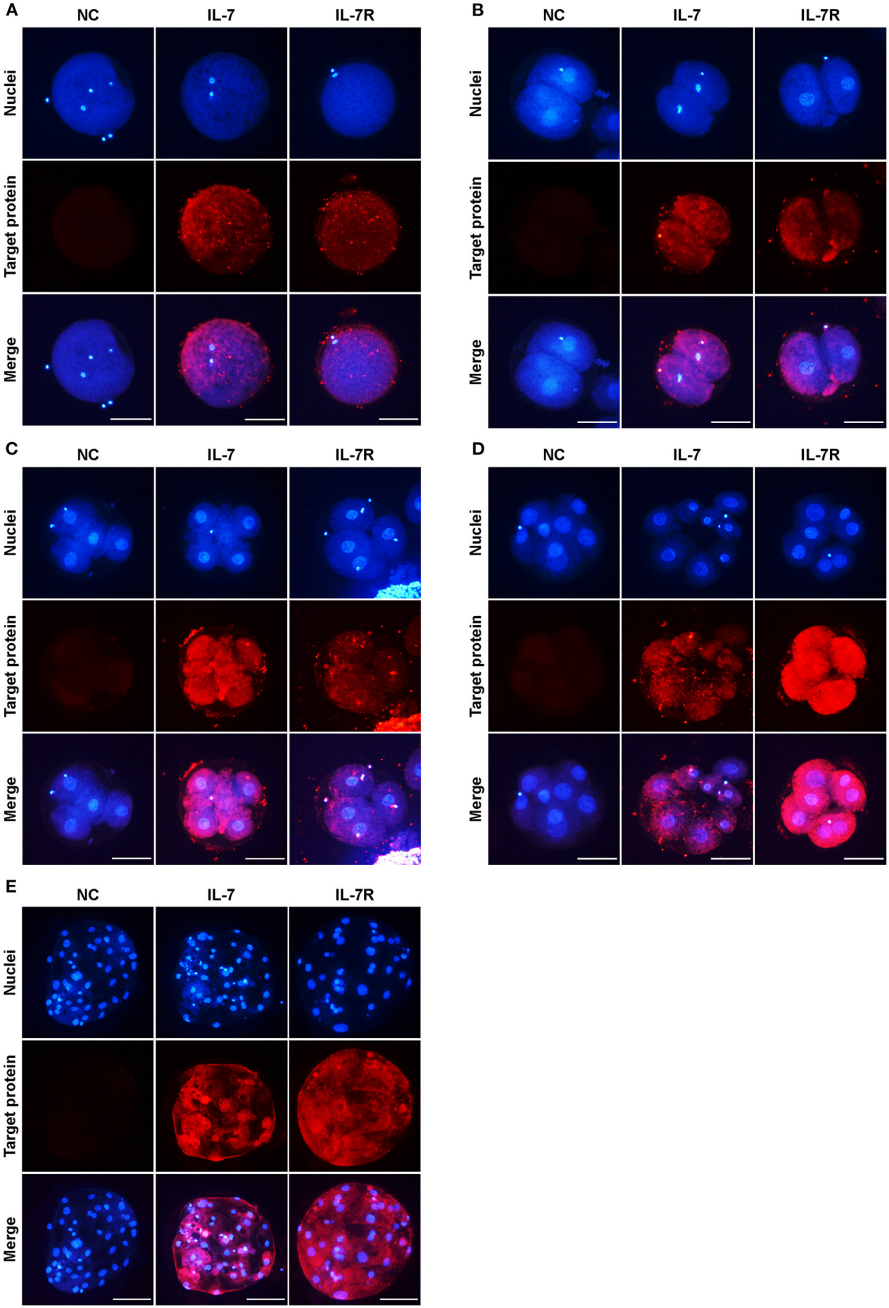
Localization of IL-7 and IL-7R in preimplantation porcine embryos. Porcine oocytes at the metaphase II–stage (MII) **(A)** and embryos at the 2- **(B)**, 4- **(C)**, 8-cell **(D)**, and blastocyst **(E)** stages collected at 0, 24, 48, 72, and 168 h after PA, respectively, were stained with antibodies against IL-7 and IL-7R. Scale bar: 100 μm.

To identify the locations of IL-7 and IL-7R in porcine oocytes and embryos at each stage, we collected MII oocytes after IVM, and sampled 2-, 4-, and 8-cell embryos and blastocysts at 24, 48, 72, and 168 h after PA, respectively. The localization of IL-7 and IL-7R proteins during preimplantation development was investigated by immunofluorescence. Both IL-7 and IL-7R were expressed in MII oocytes (Figure 1A). Furthermore, these proteins were observed in the 2-, 4-, and 8-cell stage embryos (Figures 1B–D). IL-7 and its receptor were also found to be localized to the cytoplasm until the blastocyst stage (Figure 1E).

### Effects of IL-7 supplementation during IVC on porcine embryonic development after PA

**Figure 2:**
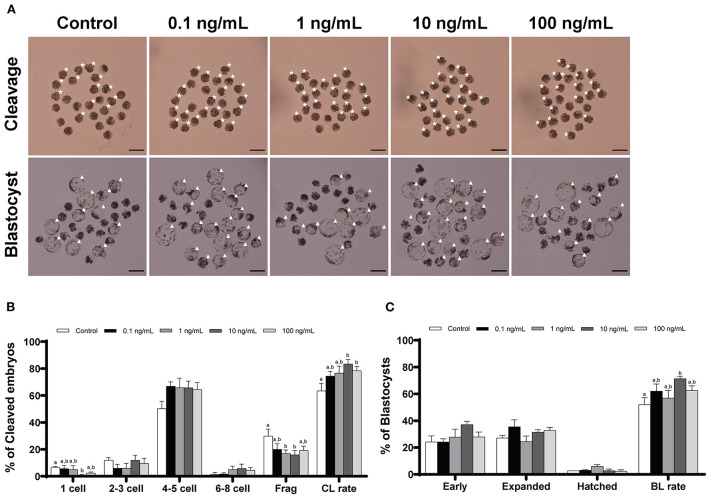
Effect of IL-7 supplementation during IVC on the cleavage and blastocyst formation patterns of PA embryos. **(A)** Representative image of the morphology of cleaved embryos and blastocysts from each group after PA. Asterisk, cleaved embryo; Triangle, blastocyst. Scale bar: 200 μm. The cleavage pattern **(B)** and blastocyst formation **(C)** rate of PA embryos. Within each endpoint, bars with different letters (a, b) indicate significant differences (*p* < 0.05) at various IL-7 concentrations. Frag, fragmentation; CL, cleavage; BL, blastocyst. The cleavage and blastocyst formation rates were evaluated on Day 2 and 7 after PA, respectively. For all graphs, values represent the mean ± SEM. The experiment was replicated four times.

We added IL-7 at various concentrations during IVC to determine its optimal concentration for porcine embryonic development after PA. The cleavage rates were significantly increased (*p* < 0.05) in the 10 and 100 ng/mL IL-7-treated groups compared to the control group (Table 1 and Figure 2). Furthermore, blastocyst formation rates were significantly higher (*p* < 0.05) in the group treated with 10 ng/mL IL-7 than in the control group. The cleavage patterns on day 2 also revealed that the rates of fragmented embryos were significantly lower (*p* < 0.05) in the 1 and 10 ng/mL IL-7-treated groups than in the control group (Figure 2B).

**Table 1:** Effect of IL-7 supplementation during IVC on embryonic development after PA.

**IL-7 concentration (ng/mL)**	No. of embryos cultured, N*	**No. (%) of embryos developed to**			
		≥**2-Cell**		**Blastocyst**	
0	135	87	(63.4 ± 5.6)^a^	71	(51.9 ± 5.2)^a^
0.1	134	99	(74.4 ± 3.5)^a^^,^ ^b^	82	(62.0 ± 5.4)^a^^,^ ^b^
1	133	102	(76.7 ± 5.1)^a^^,^ ^b^	77	(56.8 ± 5.6)^a^^,^ ^b^
10	135	112	(83.4 ± 3.3)^b^	96	(71.3 ± 1.8)^b^
100	133	105	(78.4 ± 3.2)^b^	84	(62.5 ± 3.6)^a^^,^ ^b^

N^*^: Four times replicated.

a, b Values with different superscripts within a column differ significantly (*p* < 0.05).

### Effects of IL-7 supplementation during IVC on intracellular levels of GSH and ROS in cleaved embryos

**Figure 3:**
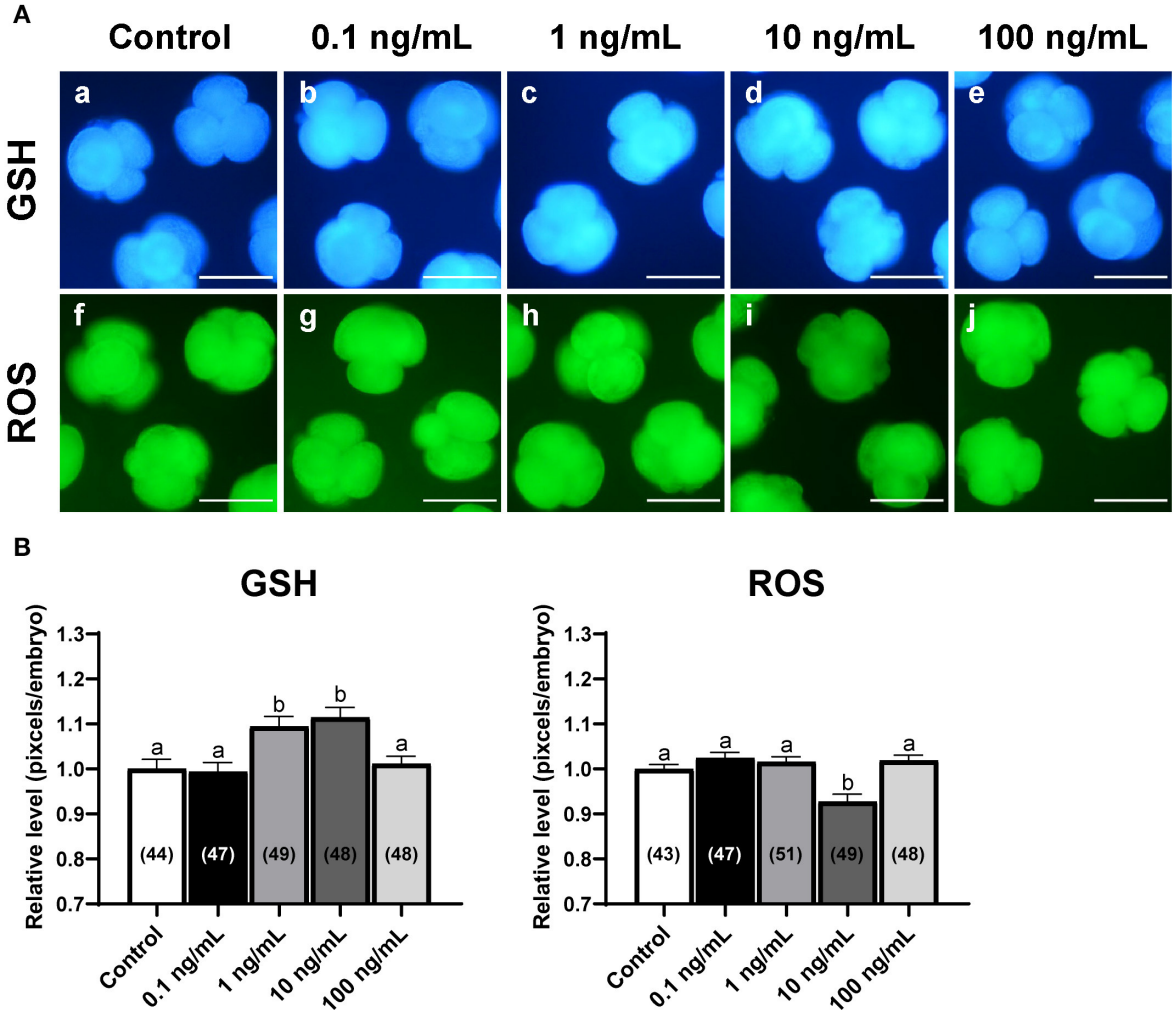
Epifluorescence photomicrographs of PA-derived 4-cell embryos with various concentrations of IL-7 supplementation during IVC for 2 days. **(A)** Embryos were stained with Cell Tracker Blue (a–e) and H_2_DCFDA (f–j) to detect intracellular levels of glutathione (GSH) and reactive oxygen species (ROS), respectively. Scale bar: 100 μm. **(B)** The relative levels of intracellular GSH and ROS levels within *in vitro* cultured porcine embryos treated with IL-7 during IVC. The number of embryos examined in each experimental group is shown in parentheses. Within each endpoint, bars with different letters (a,b) are significantly different (*p* < 0.05) for each group. For all graphs, the values represent the mean ± SEM. The experiment was replicated four times.

To examine the effect of IL-7 on oxidative stress, we analyzed intracellular GSH and ROS levels in 4-cell embryos on day 2 after PA (Figure 3). The 1 and 10 ng/mL IL-7 treatment groups showed significantly higher (*p* < 0.05) intracellular GSH levels than the control group. Further, embryos in the 10 ng/mL IL-7 treatment group showed significantly lower (*p* < 0.05) intracellular ROS levels than those in the control group.

### Effects of IL-7 supplementation during IVC on total cell number and apoptosis in blastocysts

**Figure 4:**
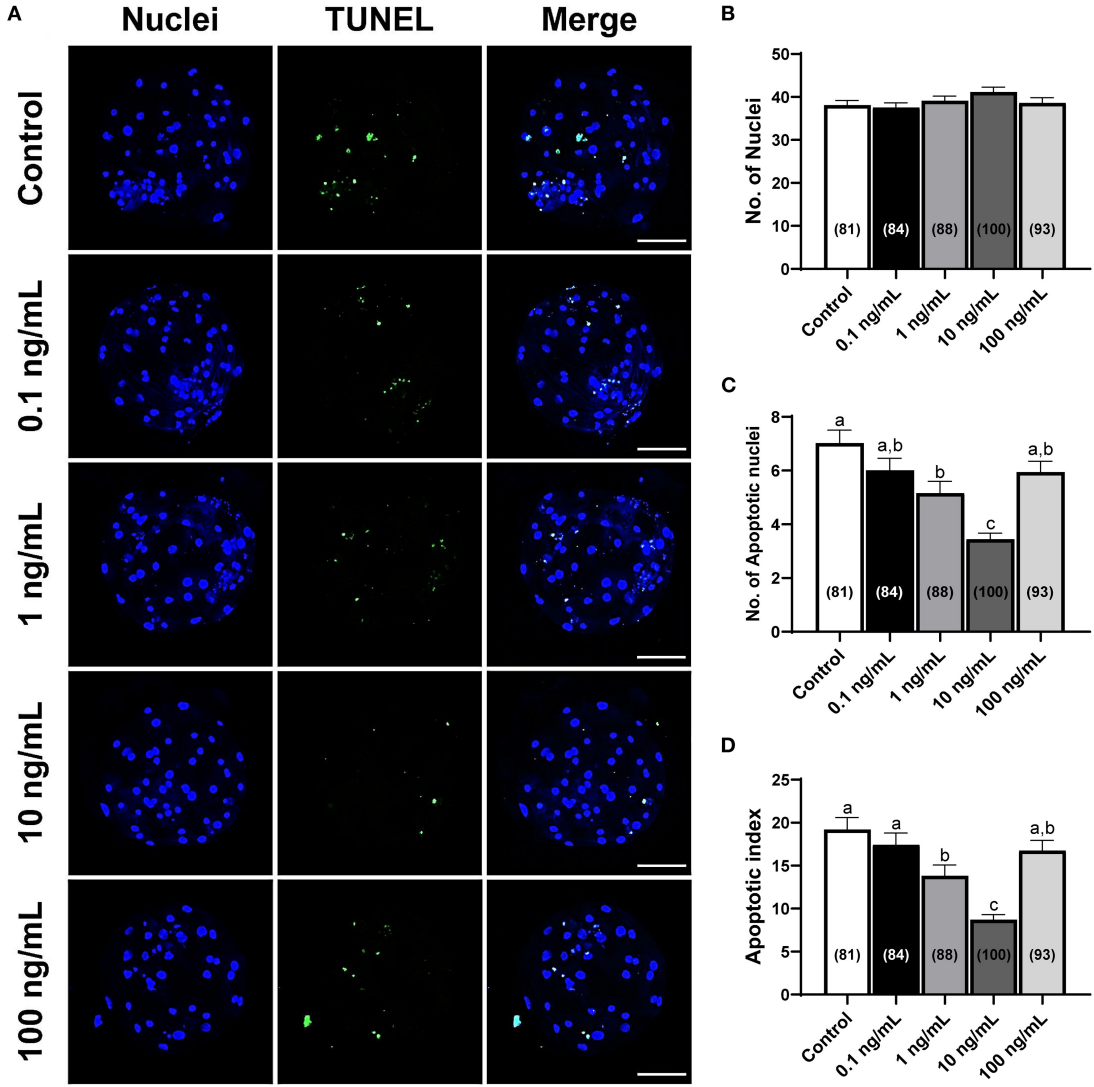
Numbers of total cells and apoptotic nuclei in PA-derived blastocysts exposed to various concentrations of IL-7 supplementation during IVC for 7 days. **(A)** Representative laser scanning confocal microscopy images (200×) of porcine PA blastocysts labeled with Hoechst 33342 (Total nuclei, blue) and TUNEL (Apoptotic nuclei, green) after IVC. Scale bar: 100 μm. **(B–D)** Quantification of total and apoptotic cell numbers, and apoptotic index in the indicated groups. The number of embryos examined in each experimental group is shown in parentheses. Within each endpoint, bars with different letters (a–c) are significantly different (*p* < 0.05) for each group. For all graphs, the values represent the mean ± SEM. The experiment was replicated four times.

The total number of cells and apoptotic nuclei were counted to investigate the quality of porcine blastocysts treated with IL-7. Supplementing the IVC media with IL-7 did not influence the total number of nuclei compared with that in the control group (Figures 4A,B). Nevertheless, the number of apoptotic nuclei and the apoptotic index were significantly reduced (*p* < 0.05) in blastocysts treated with 1 and 10 ng/mL IL-7 compared to those in the control (Figures 4A,C,D).

### Effects of IL-7 supplementation during IVC on gene expression levels in cleaved embryos and blastocysts

**Figure 5:**
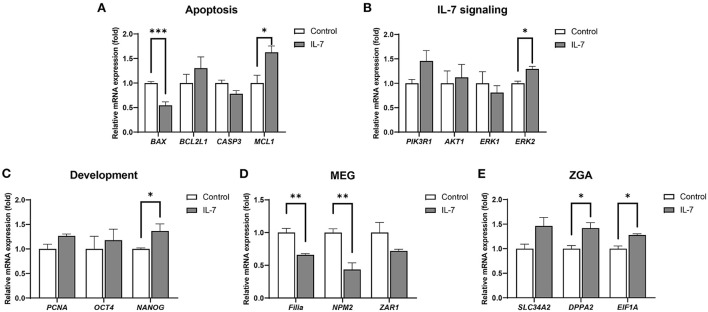
Relative mRNA expression levels of genes associated with apoptosis, IL-7 signaling, development, maternal effect genes (MEGs), and zygotic genome activation (ZGA) in IL-7-treated day 2 embryos. The mRNA expression levels of **(A)** apoptosis-related genes (*BAX, BCL2L1, CASP3*, and *MCL1*), **(B)** IL-7 signaling-related genes (*PIK3R1, AKT1, ERK1*, and ERK2), **(C)** Development-related genes (*PCNA, OCT4*, and *NANOG*), **(D)** MEGs (*Filia, NPM2*, and *ZAR1*), and **(E)** ZGA-related genes (*SLC34A2, DPPA2*, and *EIF1A*) in day 2 embryos from control and 10 ng/mL IL-7 treatment group. Data were normalized to the *RN18S* gene. Asterisks indicate statistical significance (*p < 0.05, **p < 0.01, and ***p < 0.001). For all graphs, the value represents mean ± SEM. The experiments were replicated three or four times.

After determining the optimal IL-7 supplementation concentration during the IVC period to be 10 ng/mL, we conducted subsequent IVC experiments using 10 ng/mL IL-7. To determine the mechanism of the observed increase in embryonic developmental efficiency upon IL-7 supplementation, transcript levels in day 2 embryos and blastocysts were analyzed *via* qRT-PCR. Thus, we observed that in the IL-7-treated group relative to the control group, the mRNA level of the pro-apoptotic gene, *BAX* was significantly decreased (*p* < 0.001), while that of the anti-apoptotic gene, *MCL1* was significantly increased (*p* < 0.05) (Figure 5A). The expression levels of genes associated with IL-7 signaling, including *PIK3R1, AKT1*, and *ERK1*, were not significantly different between the IL-7 supplementation and control groups. However, *ERK2* levels were significantly higher (*p* < 0.05) for the IL-7-supplemented group relative to the control group (Figure 5B). Furthermore, the IL-7-treated embryos showed significantly higher (*p* < 0.05) *NANOG* transcript levels than the control embryos (Figure 5C).

A previous study revealed that porcine ZGA occurs at the 4-cell stage (30). It has also been reported that during the maternal-to-zygotic transition (MZT), perturbation in the degradation of maternal RNAs and ZGA can lead to embryonic arrest, indicating the importance of these processes (31). Therefore, we investigated whether IL-7 is involved in maternal mRNA clearance and ZGA by detecting the mRNA levels of MEGs (*Filia, NPM2*, and *ZAR1*) and ZGA-related genes (*SLC34A2, DPPA2*, and *EIF1A*). Compared with the control group, *Filia* and *NPM2* transcript levels were significantly lower (*p* < 0.01) following IL-7 treatment (Figure 5D). Conversely, the mRNA expression levels of *DPPA2* and *EIF1A* were significantly increased (*p* < 0.05) on day 2 after the embryos were treated with IL-7 (Figure 5E).

**Figure 6:**
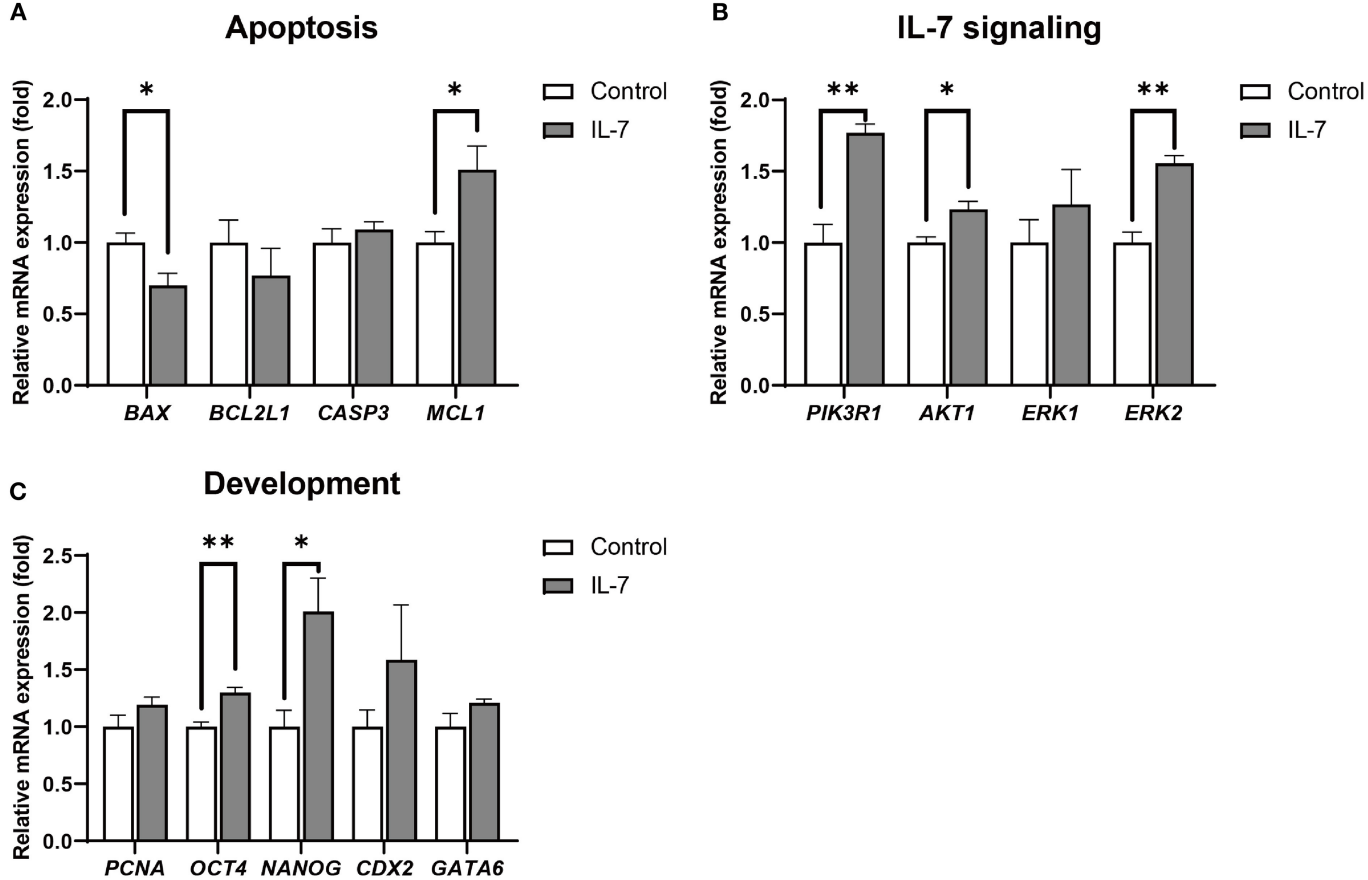
Relative mRNA expression levels of genes associated with apoptosis, IL-7 signaling, and development in IL-7-treated blastocysts. The mRNA expression levels of **(A)** apoptosis-related genes (*BAX, BCL2L1, CASP3*, and *MCL1*), **(B)** IL-7 signaling-related genes (*PIK3R1, AKT1, ERK1*, and *ERK2*) and **(C)** development-related genes (*PCNA, OCT4, NANOG, CDX2*, and *GATA6*) in blastocysts from control and 10 ng/mL IL-7 treatment group. Data were normalized to the *RN18S* gene expression. Asterisks indicate statistical significance (*p < 0.05 and **p < 0.01). For all graphs, the value represents mean ± SEM. The experiments were replicated three or four times.

Additionally, blastocysts treated with IL-7 showed significantly (*p* < 0.05) higher *MCL1* and lower *BAX* levels than the the control embryos (Figure 6A). Further, among the IL-7 signaling-related genes, the expression levels of *PI3KR1, AKT1*, and *ERK2* were significantly increased in blastocysts treated with IL-7 compared with those in the control group (Figure 6B). We also observed that the expression levels of genes related to embryonic development, *OCT4* and *NANOG*, were significantly increased (*p* < 0.01 and *p* < 0.05, respectively), while those of *CDX2, GATA6*, and *PCNA* did not show any significant difference between the control and the IL-7 treatment group (Figure 6C).

### Effects of IL-7 supplementation during IVC on pSTAT5 expression and ICM ratio of blastocysts

**Figure 7:**
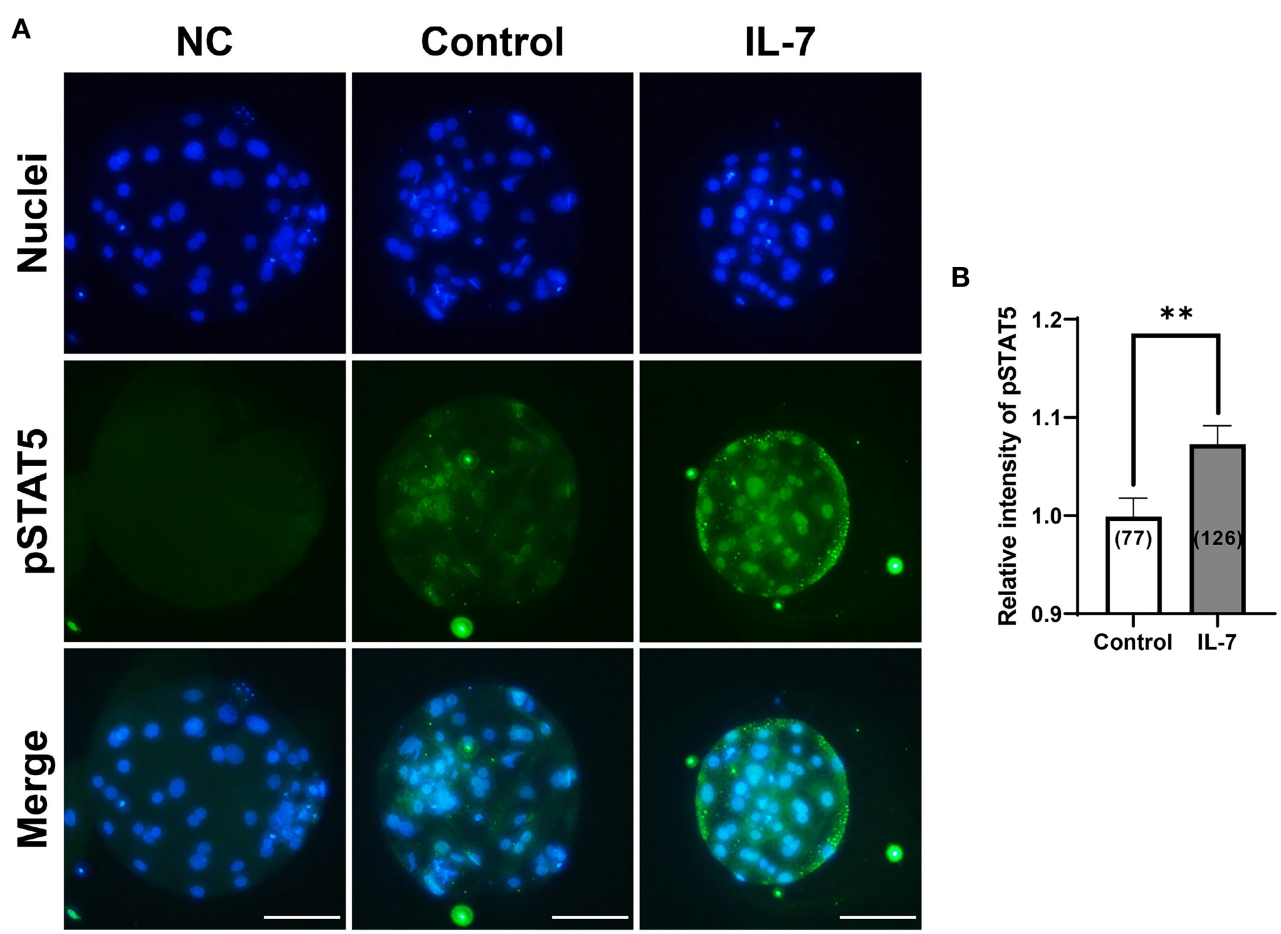
Effect of IL-7 supplementation during IVC on phosphorylated STAT5 (pSTAT5) expression of blastocysts after PA. **(A)** Representative immunofluorescence images (200×) of porcine parthenote blastocysts labeled with Hoechst 33342 (Total nuclei, blue) and pSTAT5 (green) after IVC. NC, negative control. Scale bar: 100 μm. **(B)** Quantification of the relative intensity of pSTAT5 in the indicated groups. The number of blastocysts examined in each experimental group is shown in parentheses. Asterisks indicate statistical significance (**p < 0.01). For the graph, the values represent the mean ± SEM. The experiment was replicated four times.

IL-7 activates the JAK/STAT pathway, which when stimulated leads to STAT5 phosphorylation in thymocytes and granulosa cells (32, 33). Therefore, to determine whether IL-7 supplementation during IVC stimulated the JAK/STAT pathway at the porcine blastocyst stage, we examined the expression of pSTAT5 protein *via* immunofluorescence. The pSTAT5 protein level was significantly (*p* < 0.01) increased in blastocysts treated with IL-7 compared to that in the control (Figure 7).

**Figure 8:**
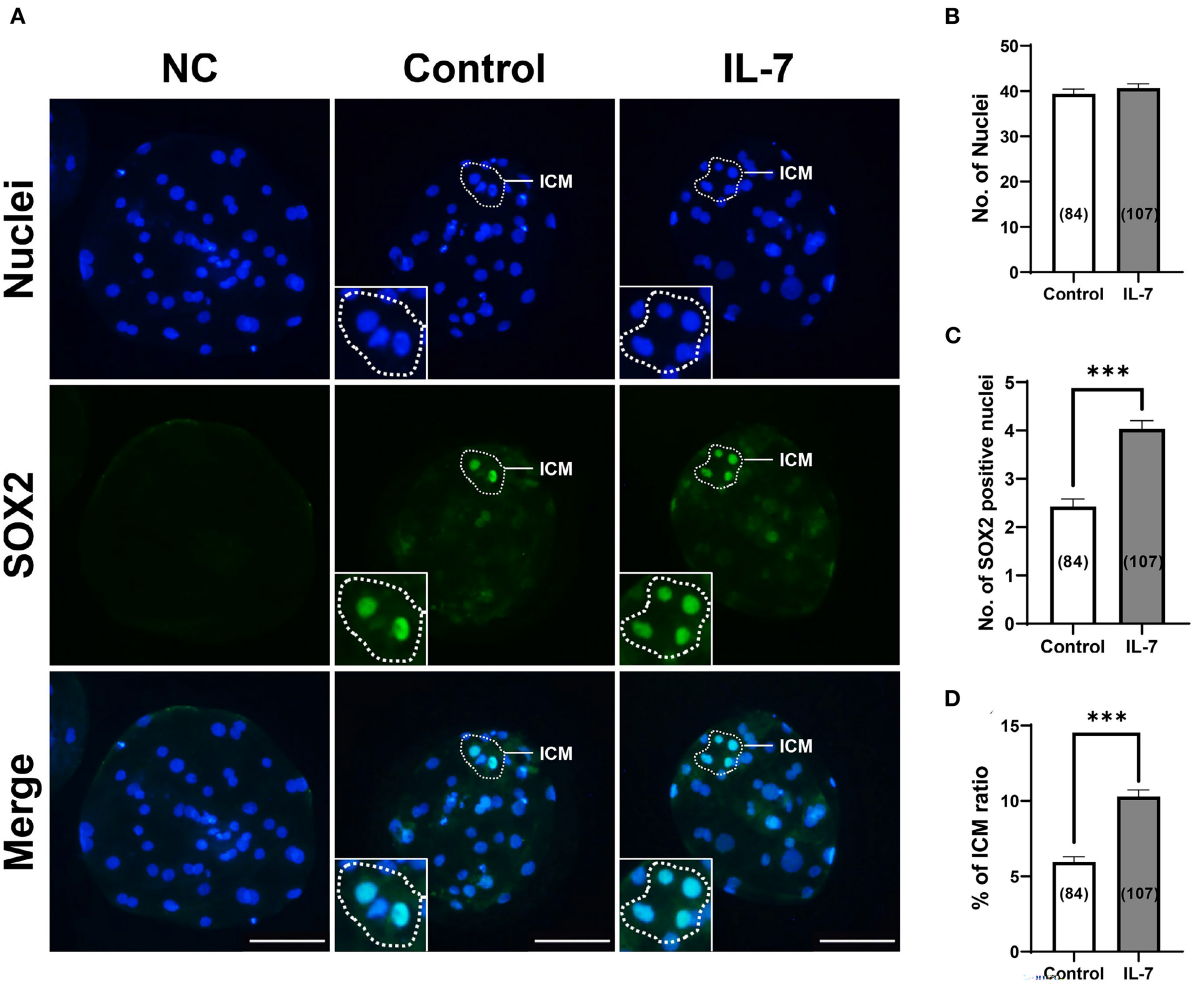
Effects of IL-7 supplementation during IVC on inner cell mass (ICM) specification of blastocysts after PA. **(A)** Representative immunofluorescence images (200×) of porcine parthenote blastocysts labeled with Hoechst 33342 (Total nuclei, blue) and SOX2 (ICM marker, green) after IVC. NC, negative control. Scale bar: 100 μm. **(B–D)** Quantification of the total, SOX2 positive cell numbers, and ICM ratio in the indicated groups. The number of embryos examined in each experimental group is shown in parentheses. Asterisks indicate statistical significance (***p < 0.001). For all graphs, the values represent the mean ± SEM. The experiment was replicated four times.

Based on qRT-PCR, IL-7-treated blastocysts showed significantly increased levels for the epiblast marker, *NANOG*, but not the trophectoderm marker, *CDX2*. Further, we hypothesized that IL-7 supplementation affects ICM development during lineage segregation in porcine blastocysts. Thus, blastocysts were stained for SOX2, a faithful ICM marker (34), to determine the effect of IL-7 on the number of ICM cells (Figure 8). Supplementation with IL-7 during the IVC period did not affect the total cell number (Figure 8B). However, embryos treated with IL-7 developed into blastocysts with significantly increased (*p* < 0.001) numbers of SOX2+ cells compared to the control (4 cells in IL-7 vs. 2.4 cells in controls) (Figure 8C). The ICM ratio was also significantly enhanced (*p* < 0.001) by ~1.7-fold in porcine blastocysts treated with IL-7 (10.3% in IL-7 vs. 5.9% in control) (Figure 8D).

## Discussion

In this study, we examined the expression of IL-7 protein as well as that of its receptor, IL-7R in preimplantation porcine embryos. In addition, we demonstrated the optimal concentration of IL-7 in an *in vitro* embryo culture experiment, which increased the cleavage and blastocyst formation rate by regulating apoptosis-, IL-7 signaling-, and development-related genes. Further, we demonstrated that IL-7 supplementation reduced ROS levels as well as the transcript levels of MEG-related genes. It also improved GSH levels and the mRNA expression levels of ZGA-related genes in cleaved embryos. Additionally, IL-7 supplementation also enhanced the expression of pSTAT5 protein and ICM marker, SOX2, suggesting that this is a mechanism by which IL-7 signaling modulates the development of porcine embryos *in vitro*.

Early embryonic development in domestic animals is thought to occur in the uterus following blastocyst implantation after fertilization (35). Several studies have also shown that cytokines, such as ILs, colony-stimulating factors, transforming growth factors, and tumor necrosis factors, are important for early embryonic development and implantation (14, 36, 37). Although several types of ILs exist, there is limited information regarding the role of IL-7 in preimplantation embryonic development. In a mouse study, the mRNA expression of IL-7 reportedly varied at different stages of embryonic development (38). Recently, it was also reported that IL-7 and IL-15, which belong to the same IL-2 superfamily, show highly increased gene expression in porcine reproductive tissues compared with other IL-type cytokines (39). Consistent with this previously reported finding, we detected IL-7 and its receptor IL-7R in porcine preimplantation embryos. Therefore, IL-7 probably plays an important role in porcine embryonic development.

Additionally, another previous study showed that the expression of IL-7, an oocyte-secreted factor that mediates oocyte-to-somatic cell signals, was considerably activated by meiotic progression. IL-7 was also identified as a potential candidate for oocyte quality screening tests (22). Recently as well, it was demonstrated that IL-7 improves oocyte maturation and developmental potential *via* the regulation of apoptosis-related genes when administered within an IVM system for sheep (40) and pigs (21). Thus, we speculated that IL-7 possibly plays a similar role in porcine IVC, and in this regard, our results demonstrated that IL-7 treatment favored the *in vitro* development of porcine preimplantation embryos after PA, and reduced fragmentation in day 2 embryos. Furthermore, the number of apoptotic cells was reduced and pSTAT5 protein levels were increased in blastocysts treated with IL-7. Reportedly, the phosphorylated STAT5 induced by IL-7 translocates to the nucleus, where it regulates the expression of various genes involved in the prevention of apoptosis (41–43). Additionally, in lymphoid development, IL-7 improved the survival of immature thymocytes and mature T cells by increasing the level of the anti-apoptotic protein MCL1, thereby inhibiting BAX and BAK activation (44). Consistently, our results showed that the mRNA expression levels of *MCL1* significantly increased, while that of *BAX* decreased in both IL-7-treated day 2 embryos and blastocysts, suggesting that IL-7 enhances the *in vitro* development of porcine embryos by preventing them from undergoing apoptosis. However, further studies are needed to evaluate the protein levels of these apoptosis-related genes and the IL-7/STAT5 pathway in porcine IVC.

The early embryonic development of domestic animals is regulated primarily by maternal mRNA and proteins. Subsequently, the maternally deposited transcripts become progressively degraded during the MZT, and this is accompanied by ZGA (45). It was also been observed that ZGA defects lead to the abnormal clearance of maternal mRNAs during the human MZT process, thereby retarding embryonic development (46). Therefore, ZGA plays an important role in early embryonic development (47). The results of qRT-PCR analyses in this study showed that day 2 embryos treated with IL-7 exhibited increased maternal mRNA degradation (*Filia* and *NPM2*), while the mRNA expression levels of ZGA-related genes (*DPPA2* and *EIF1A*) were enhanced. Additionally, *NANOG* transcript levels were significantly higher in day 2 embryos treated with IL-7 than in the control group. The results of a previous study showed that Nanog and Pou5f1 (OCT4) regulate ZGA expression in zebrafish (48). This observation indicated that IL-7 plays an important role in porcine embryonic development by modulating maternal mRNA clearance and ZGA.

In domestic animals, oxidative stress during preimplantation development can lead to developmental arrest at the time of ZGA or blastocyst apoptosis (49). In fact, exogenous and endogenous oxidative stress during *in vitro* embryo manipulation reportedly contributes to developmental arrest (50). Although ROS release due to oxidative stress is considered an unwanted oxidative product, small amounts of ROS can be utilized in physiological signaling pathways in embryos (51). It has also been observed that a moderate concentration of IL-7 during IVM increases ROS levels in ovine oocytes (40). In one of our previous studies, we performed an experiment using a porcine IVM system to determine whether IL-7 treatment regulates oxidative stress (21). Consistent with the results of the present study, we observed that IL-7 supplementation during porcine IVC increased intracellular GSH levels and reduced ROS levels in 4-cell embryos, provoking ZGA. In mammalian embryos, increased ROS levels have been observed in ZGA, which is sensitive to oxidative stress during embryonic development (49, 52). Furthermore, this peak in ROS level during ZGA indicated that oxidant defenses can be depleted in the MZT and must be replenished by zygotic gene expression (53). Collectively, our results in this study suggested that IL-7 reduces oxidative stress by regulating ZGA-related genes during porcine MZT.

In domestic animals, two major events result in lineage segregation during the preimplantation development. First, during the compacted morula stage, the trophectoderm is formed. This is in turn involved in the formation of placenta and ICM, which becomes the definitive structure of the fetus and can produce ESCs. Second, ICM is divided into the epiblast and primitive endoderm (54). Recent advances in single blastomere transcriptomics have facilitated the elucidation of the signaling pathways involved in lineage specification of preimplantation embryos in mammals (30, 55–58). Reportedly, *CDX2* is a trophectodermal marker in mice, humans, and pigs (54, 59), and *OCT4*, a core pluripotency gene, is expressed in all preimplantation embryos. Additionally, the epiblast marker, *NANOG*, and the hypoblast marker, *GATA6*, are co-expressed in the ICM of porcine early blastocysts (55, 56). In the present study, we showed that in blastocysts, IL-7 supplementation significantly enhanced the expression of *OCT4* and *NANOG* mRNA, but not *CDX2* or *GATA6* mRNA. These pluripotency-related genes are regulated *via* an indirect pathway involving other IL-type cytokines (60, 61). However, there are no reports showing that IL-7 regulates pluripotency genes. Therefore, further studies are needed to verify these findings.

Reportedly, IL-7 activates the JAK/STAT, PI3K/Akt, and mitogen-activated protein kinase/extracellular signal-regulated kinase (MAPK/ERK; MEK) pathways in normal T cells and T-cell acute lymphoblastic leukemia cells (33, 62–64). Consistent with these previous studies, our results also showed increased pSTAT5 protein expression as well as the upregulation of PI3K/Akt pathway-related genes and *ERK2* in IL-7-treated blastocysts. The PI3K/Akt pathway plays a functional role in preimplantation embryos, and its inhibition influences development to the blastocyst stage in murine, bovine, and porcine embryos (65–67). Further, ERK signaling activation plays an essential role in ICM differentiation in murine blastocysts (68). The function of these pathways is enhanced in the ICM of porcine blastocysts and can affect the number of ICM cells when disrupted by specific pathway inhibitors (55, 56). Similarly, our results showed that IL-7 treatment enhanced the number of ICM cells and the ICM ratio of blastocysts, suggesting that IL-7 enhances blastocyst quality by activating IL-7 signaling during porcine embryonic development. Consistent with previously reported results, an increase in the number of ICM cells was observed when other IL types were also administered during the IVC period (69–72). Notwithstanding, further studies are required to determine the exact role of IL-7 in porcine ICM development.

## Conclusion

In this study, we demonstrated, for the first time, the localization of IL-7 and IL-7R in preimplantation porcine embryos. Specifically, our results showed that IL-7 and its receptor are involved in porcine embryonic development and that IL-7 supplementation in porcine IVC enhances the development of porcine parthenote embryos *via* the regulation of the anti-apoptotic effect, maternal mRNA clearance, and ZGA. Further, we observed that IL-7 supplementation improved blastocyst quality by modulating the transcription of development- and IL-7 signaling-related genes and by increasing the number of SOX2+ cells and the ICM ratio during porcine PA embryo development *in vitro*. In this study, we focused on the effect of IL-7 on porcine embryonic development using only PA embryos, excluding male counterpart factors that can cause problems, such as polyspermy. However, given that there are actually many mechanistic differences between PA and IVF-derived embryos, further studies using IVF or somatic cell nuclear transfer-derived embryos are required. Our findings, primarily, provide new insights into the effects of IL-7 on embryonic development and may contribute to the improvement of porcine IVC systems and related technologies.

## Data availability statement

The original contributions presented in the study are included in the article/Supplementary material, further inquiries can be directed to the corresponding authors.

## Author contributions

DO, YJ, and S-HH: conceptualization, validation, writing-original draft preparation, and writing-review and editing. DO, HC, MK, LC, JL, GL, and YJ: methodology. DO, HC, MK, LC, JL, AJ, SK, and HZ: investigation. DO, HC, MK, LC, JL, and GL: formal analysis. S-HH: funding acquisition. All authors have read and agreed to the published version of the manuscript.
