# Supplementary material for: Interleukin-7 enhances in vitro development and blastocyst quality in porcine parthenogenetic embryos
Source: Front Vet Sci. 2022 Dec 8;9:1052856. doi: 10.3389/fvets.2022.1052856 (PMC9772438; doi:10.3389/fvets.2022.1052856)
Supplement: Supplementary file 1 [file Table_1.DOCX]

Supplementary Material

# Supplementary Table 1

# Primer sequences used for qRT-PCR.

| **mRNA** | **Primer sequences** | **Product size (bp)** | **GenBank**  **accession number** |
| --- | --- | --- | --- |
| *RN18S* | F: 5'-CGCGGTTCTATTTTGTTGGT-3' | 219 | NR_046261.1 |
|  | R: 5'-AGTCGGCATCGTTTATGGTC-3' |  |  |
| *BAX* | F: 5'-TGCCTCAGGATGCATCTACC-3' | 199 | XM_013998624.2 |
|  | R: 5'-AAGTAGAAAAGCGCGACCAC-3' |  |  |
| *BCL2L1* | F: 5'-AATGACCACCTAGAGCCTTG-3' | 182 | NM_214285.1 |
|  | R: 5'-GGTCATTTCCGACTGAAGAG-3' |  |  |
| *CASP3* | F: 5'-CGTGCTTCTAAGCCATGGTG-3' | 186 | NM_214131.1 |
|  | R: 5'-GTCCCACTGTCCGTCTCAAT-3' |  |  |
| *MCL1* | F: 5'-GGACATCAAAAACGAAGACG-3' | 181 | NM_001348806.1 |
|  | R: 5'-TGTGATGCTTTCTGCTAACG-3' |  |  |
| *PIK3R1* | F: 5'-CCACTACCGGAATGAATCTC-3' | 211 | XM_021076847.1 |
|  | R: 5'-TTCCTGGGAAGTACGGGTAT-3' |  |  |
| *AKT1* | F: 5'-CTACAACCAGGACCACGAGA-3' | 208 | NM_001159776.1 |
|  | R: 5'-CTCATACACATCCTGCCACA-3' |  |  |
| *ERK1* | F: 5'-ATCACAGTGGAGGAAGCACT-3' | 202 | XM_021088019.1 |
|  | R: 5'-GAGGCATCTGTCCAGGTTAG-3' |  |  |
| *ERK2* | F: 5'-AGTCCATCGACATCTGGTCT-3' | 240 | NM_001198922.1 |
|  | R: 5'-GAGCTTTGGAGTCAGCATTT-3' |  |  |
| *PCNA* | F: 5'-CCTGTGCAAAAGATGGAGTG-3' | 187 | NM_001291925.1 |
|  | R: 5'-GGAGAGAGTGGAGTGGCTTTT-3' |  |  |
| *OCT4* | F: 5'-GCGGACAAGTATCGAGAACC-3' | 200 | NM_001113060.1 |
|  | R: 5'-CCTCAAAATCCTCTCGTTGC-3' |  |  |
| *NANOG* | F: 5'-TAAAACCACTGCCCACATCT-3' | 131 | NM_001129971.1 |
|  | R: 5'-CTGCCTCTGAAATCTGTCGT-3' |  |  |
| *CDX2* | F: 5'-CTGTTTGGGTTGTTGGTCTG-3' | 95 | NM_001278769.1 |
|  | R: 5'-CCCACTCCCTTCACCATATC-3' |  |  |
| *GATA6* | F: 5'-GAGGGAATTCAGACCAGGAA-3' | 159 | NM_214328.2 |
|  | R: 5'-AGCTGGCGTTTGTGTTGTAG-3' |  |  |
| *Filia* | F: 5'-GCCCTACTGGTTTCACTCAG-3' | 193 | XM_021089534.1 |
|  | R: 5'-ATAAGGCCGTCCAAATATCA-3' |  |  |
| *NPM2* | F: 5'-GCTCTGGACCTGTGTTCCTC-3' | 220 | NM_001195362.1 |
|  | R: 5'-GCTGCACTTGTCTGCTTCTG-3' |  |  |
| *ZAR1* | F: 5'-TCCTGCCCAGTAAAACTTC-3' | 195 | NM_001129956.1 |
|  | R: 5'-AAAAAGGCTCACTTGTCTGC-3' |  |  |
| *SLC34A2* | F: 5'-CTGTGTCTTCCAAGGGATTG-3' | 197 | NM_001256772.1 |
|  | R: 5'-GAATCAGGACTGTCACCAAC-3' |  |  |
| *DPPA2* | F: 5'-ACCAGATTGCAGTCAGCTTC-3' | 185 | XM_003358822.4 |
|  | R: 5'-TTCACAGCCTTAGGGTGAAC-3' |  |  |
| *EIF1A* | F: 5'-CTGGGAAATGGACGATTAGA-3' | 198 | NM_001243218.1 |
|  | R: 5'-GCCATATGCCTTCAGACTTC-3' |  |  |
